# Integration of FTIR Spectroscopy, Volatile Compound Profiling, and Chemometric Techniques for Advanced Geographical and Varietal Analysis of Moroccan Eucalyptus Essential Oils

**DOI:** 10.3390/s24227337

**Published:** 2024-11-17

**Authors:** Aimen El Orche, Abdennacer El Mrabet, Amal Ait Haj Said, Soumaya Mousannif, Omar Elhamdaoui, Siddique Akber Ansari, Hamad M. Alkahtani, Shoeb Anwar Ansari, Ibrahim Sbai El Otmani, Mustapha Bouatia

**Affiliations:** 1 Laboratory of Drugs Sciences, Biomedical Research and Biotechnology, Faculty of Medicine and Pharmacy, Hassan II University of Casablanca, B.P. 9154, Casablanca 20250, Morocco; abdennacer.elmrabet-etu@etu.univh2c.ma (A.E.M.); amal.aithaj@gmail.com (A.A.H.S.); sbai.ibrahim@gmail.com (I.S.E.O.); 2 Laboratory of Analytical Chemistry, Team of Formulation and Quality Control of Health Products, Faculty of Medicine and Pharmacy, Mohammed V University in Rabat, Rabat 10090, Morocco; mousannif.soumaya@gmail.com (S.M.); omar.elhamdaoui@um5s.net.ma (O.E.); m.bouatia@um5r.ac.ma (M.B.); 3 Pharmacology and Toxicology Laboratory, Faculty of Medicine and Pharmacy, Cadi Ayyad University, Marrakech 40000, Morocco; 4 Department of Pharmaceutical Chemistry, College of Pharmacy, King Saud University, P.O. Box 2457, Riyadh 11451, Saudi Arabia; sansari@ksu.edu.sa (S.A.A.); ahamad@ksu.edu.sa (H.M.A.); 5 Department of Drug Science and Technology, University of Turin, 10124 Turin, Italy; shoeb.ansari@edu.unito.it

**Keywords:** eucalyptus essential oil, FTIR Spectroscopy, volatile compound profiles, chemical composition variations, chemometric analyses

## Abstract

Eucalyptus essential oil is widely valued for its therapeutic properties and extensive commercial applications, with its chemical composition significantly influenced by species variety, geographical origin, and environmental conditions. This study aims to develop a reliable method for identifying the geographical origin and variety of eucalyptus oil samples through the application of advanced analytical techniques combined with chemometric methods. Essential oils from Eucalyptus globulus and Eucalyptus camaldulensis were analyzed using Gas Chromatography–Flame Ionization Detection (GC–FID) and Fourier Transform Infrared (FTIR) Spectroscopy. Chemometric analyses, including Orthogonal Partial Least Squares-Discriminant Analysis (O2PLS-DA) and Hierarchical Cluster Analysis (HCA), were utilized to classify the oils based on their volatile compound profiles. Notably, O2PLS-DA was applied directly to the raw FTIR data without additional spectral processing, showcasing its robustness in handling unprocessed data. For geographical origin determination, the GC–FID model achieved a Correct Classification Rate (CCR) of 100%, with 100% specificity and 100% sensitivity for both calibration and validation sets. FTIR spectroscopy achieved a CCR of 100%, specificity of 100%, and sensitivity of 100% for the calibration set, while the validation set yielded a CCR of 95.83%, specificity of 99.02%, and sensitivity of 94.44%. In contrast, the analysis based on species variety demonstrated 100% accuracy across all metrics CCR, specificity, and sensitivity—for both calibration and validation using both techniques. These findings underscore the effectiveness of volatile and infrared spectroscopy profiling for quality control and authentication, providing robust tools for ensuring the consistency and reliability of eucalyptus essential oils in various industrial and therapeutic applications.

## 1. Introduction

Eucalyptus oil, derived from the leaves of various Eucalyptus species, is celebrated for its diverse therapeutic and commercial applications. It possesses antimicrobial, anti-inflammatory, analgesic, and antioxidant properties, making it a valuable component in pharmaceuticals, aromatherapy, cosmetics, and the food industry [1]. Its widespread use ranges from treating respiratory ailments and muscle pain to serving as an active ingredient in skincare products and food preservatives [2].

The chemical composition of eucalyptus oil is influenced by several factors, including species variety, geographical origin, and environmental conditions [3]. These factors lead to variations in the oil’s volatile compounds, which directly impact its quality, efficacy, and specific applications [3,4]. For instance, the concentration of key components like 1,8-cineole, alpha-pinene, and limonene can vary significantly, affecting the oil’s therapeutic properties and market value [5].

Understanding the geographical and varietal origin of eucalyptus oil is crucial for several reasons. Firstly, it ensures quality control and standardization, which are essential for maintaining the therapeutic efficacy of pharmaceutical products [6]. Secondly, it aids in the authentication of eucalyptus oil, protecting consumers from adulteration and ensuring they receive genuine products with consistent quality [7]. Lastly, knowledge of the origin helps in optimizing cultivation practices to produce high-quality oil with desired properties, benefiting both producers and end-users [7].

Despite the wide-ranging applications and importance of eucalyptus oil, the identification of its volatile components, as well as the differentiation of its varieties and geographical origins, has posed significant challenges. Traditional analytical methods, such as simple chemical assays, have often been insufficient in distinguishing subtle variations in volatile profiles, which are critical for the oil’s quality and effectiveness. Recent advancements in analytical techniques, both domestically and internationally, have made significant strides in overcoming these challenges.

Globally, researchers have applied advanced techniques like Gas Chromatography (GC) and Fourier Transform Infrared (FTIR) spectroscopy, often combined with chemometric approaches, to more accurately identify the volatile compositions and differentiate eucalyptus oil by species and origin [8,9,10,11]. For instance, studies in Australia and Brazil, where eucalyptus is widely cultivated, have successfully utilized GC–MS and FTIR to analyze the oils, highlighting the impact of environmental conditions on the chemical composition of different species [12]. In Morocco, where eucalyptus oil production is expanding, similar methods have begun to show promise in differentiating local varieties and ensuring authenticity in the market [13]. However, challenges such as variability in climatic conditions, soil composition, and cultivation practices continue to affect the reproducibility and robustness of these analytical methods. Furthermore, the existing techniques sometimes struggle with the complexity of volatile compounds, necessitating the integration of more sophisticated chemometric models to better analyze and interpret the data.

When combined with chemometric analysis, these techniques can uncover complex patterns and relationships within the data, enabling the differentiation of eucalyptus oil based on geographical origin and variety [14,15,16]. Chemometrics applies mathematical and statistical methods to interpret the large datasets generated by GC–FID and FTIR, enhancing our understanding of the oil’s composition and its influencing factors.

This study presents a novel approach by integrating Gas Chromatography with Flame Ionization Detection (GC–FID) and Fourier Transform Infrared (FTIR) spectroscopy with advanced chemometric techniques, such as OPLS-DA, and HCA, to analyze the volatile compounds and infrared spectra of Moroccan Eucalyptus essential oils. The primary objective is to develop a reliable method for identifying the geographical origin and variety of eucalyptus oil samples. This innovative combination enhances the accuracy and robustness of the analysis, ensuring superior quality control, authentication, and standardization. The methodology supports the efficacy and safety of eucalyptus oil for pharmaceutical and other applications, addressing critical industry needs and contributing to scientific knowledge by setting a new precedent for essential oil analysis.

## 2. Materials and Methods

### 2.1. Essential Oil Samples

**Figure 1:**
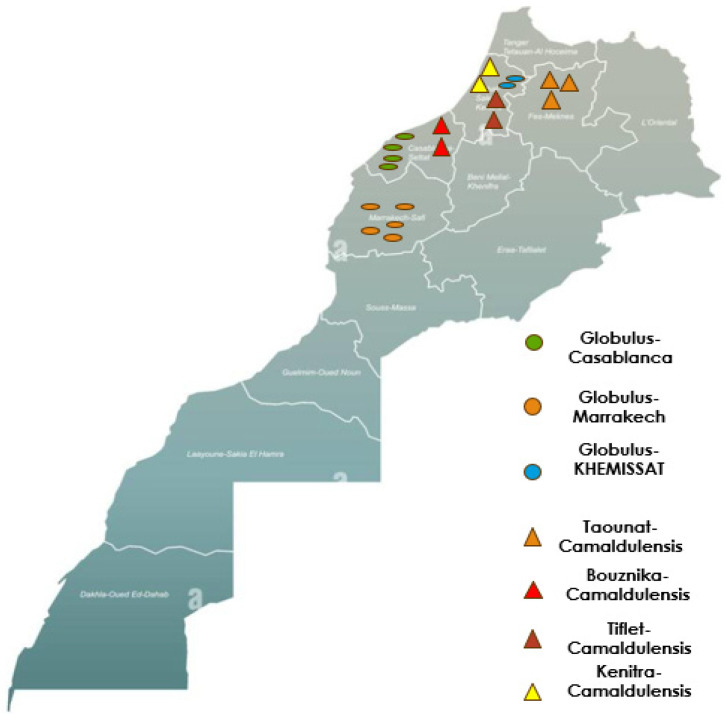
The geographical positions of the samples.

The eucalyptus oil samples in this study were obtained from fresh, healthy leaves of two cultivars, *Eucalyptus globulus* and *Eucalyptus camaldulensis*. Essential oils were extracted by hydrodistillation using a “Clevenger” apparatus. For each test, 200 g of plant material was processed. Leaves were collected from individual trees that were widely dispersed within the same orchard, as well as from trees grown in different orchards across various regions of Morocco between September and October 2022. The Globulus variety was sampled in Khemisset, Marrakech, and Casablanca, while the Camaldulensis variety was collected in Kenitra, Bouznika, Taounate, and Tiflet. Figure 1 shows the geographical positions on the map of Morocco at which the samples were taken.

After harvesting, the leaves were naturally dried in the shade within a well-ventilated and non-humid room. Once dried, the leaves underwent a hydro-distillation process lasting three hours at a temperature of 100 °C to extract the eucalyptus oil. The hydro-distillation was conducted using a Clevenger-based extraction apparatus, with distilled water as the extraction solvent.

For this study, a total of 79 eucalyptus essential oil samples were prepared and analyzed. The samples were divided into two sets: a calibration set consisting of 59 samples and a validation set comprising 20 samples. The calibration set was used to build and train the chemometric models, ensuring they could accurately classify the oils based on their chemical composition. The validation set was then employed to test and validate the performance of these models. Each sample was carefully prepared, with precise quantities measured and analyzed using both Gas Chromatography–Flame Ionization Detection (GC–FID) and Fourier Transform Infrared (FTIR) Spectroscopy.

### 2.2. Volatile Compound Determination

**Figure 2:**
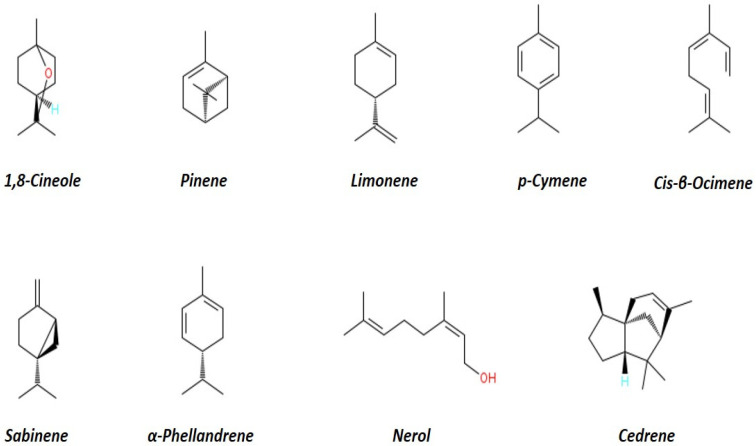
Key Constituents of Eucalyptus Leaf Essential Oils.

The essential oil samples extracted from Eucalyptus samples (Globulus and Eucalyptus camaldulensis) were prepared for comprehensive analysis to determine their volatile compound profiles. This study aims to utilize ATR-MIR spectroscopy for the initial spectrum acquisition of the samples, followed by a detailed quantitative analysis using Gas Chromatography–Flame Ionization Detection (GC–FID). The GC–FID analysis will be conducted using an Agilent 6850 GC system equipped with a Flame Ionization Detector (5301 Stevens Creek Blvd. Santa Clara, CA 95051, USA) and a 20 m × 0.1 mm DB-WAX column. Each sample, injected at 0.2 microliters, will undergo separation of organic compounds based on their volatility, with detection performed by the FID using hydrogen as the carrier gas. The study will also calculate linear retention indices for n-alkanes (C8–C28) to aid in identifying and quantifying specific compounds within the eucalyptus oils as shown in Figure 2.

### 2.3. Spectra Acquisition by ATR-MIR

The spectral analysis of eucalyptus oil samples was conducted using an ATR-FTIR spectrometer (JASCO, Lisses (Essonne), France) in the mid-infrared region (4000–600 cm^−1^). The instrument was equipped with appropriate software for spectral acquisition and analysis. Spectra were acquired with a scan speed of 0.2 cm/s and a resolution of 4 cm^−1^. Each eucalyptus oil sample underwent 20 scans to ensure robust data collection. A background spectrum was recorded using the ATR crystal as a reference to enhance spectral quality and accuracy. Post-acquisition, the spectra were meticulously analyzed to pinpoint specific spectral regions exhibiting significant intensity variations. This approach facilitated the identification of distinctive chemical signatures and differentiation among eucalyptus oil samples sourced from diverse geographical origins and varieties.

### 2.4. Chemometric Analysis of Eucalyptus Oil

To analyze the volatile composition data of Eucalyptus essential oils, we selected Orthogonal Partial Least Squares Discriminant Analysis (O2PLSDA) and Hierarchical Cluster Analysis (HCA). O2PLSDA was chosen over PLS-DA because it offers distinct advantages in separating systematic variation into predictive and orthogonal components. This separation improves model interpretability by isolating class-correlated variation (predictive) from unrelated variation (orthogonal), which enhances the clarity of class discrimination and reduces noise in complex datasets. The volatile compound data obtained from Gas Chromatography–Flame Ionization Detection (GC–FID) was utilized without additional spectral processing. O2PLSDA models were subsequently developed and validated through cross-validation techniques. The resulting loadings and score plots identified key volatile compounds as potential biomarkers for specific Eucalyptus varieties and geographical origins.

Following this, HCA was applied to the O2PLSDA scores using Euclidean distance and Ward’s linkage method to visualize natural groupings within the data. The dendrogram constructed from HCA corroborated the O2PLSDA findings, further highlighting distinct profiles across different Eucalyptus varieties and origins. This comprehensive approach provides a deeper understanding of the chemical diversity in Eucalyptus essential oils.

Orthogonal Partial Least Squares Discriminant Analysis (O2PLSDA) extends Partial Least Squares Discriminant Analysis (PLS-DA) by incorporating an Orthogonal Signal Correction (OSC) filter, which decomposes the predictor matrix (X) into blocks of variation that are either correlated or orthogonal to the response matrix (Y) [17]. This decomposition allows O2PLSDA to handle noise and irrelevant variability more effectively than PLS-DA, making it particularly suitable for complex datasets. The predictive variation, derived through a process called ‘target rotation’, enhances model transparency and improves interpretability by clearly distinguishing between predictive (class-correlated) and non-predictive (orthogonal) variation. This feature makes O2PLSDA especially useful in fields like metabonomics, where complex and high-dimensional data are common.

Evaluating the performance of chemometrics classification models involves using several metrics to ensure accuracy, precision, and reliability. Sensitivity (or recall) measures the model’s ability to correctly identify true positives, while specificity assesses the ability to correctly identify true negatives [18,19,20,21]. The Correct Classification Rate (CCR) provides an overall accuracy measure by calculating the proportion of correctly classified instances out of the total instances. The Area Under the Receiver Operating Characteristic Curve (AUC-ROC) is another critical metric, offering a comprehensive view of the model’s discriminatory power across various threshold levels. By utilizing these metrics, researchers can thoroughly assess the effectiveness and robustness of their classification models, ensuring they can confidently differentiate between classes, such as distinguishing between different geographical origins or varieties of essential oils. Rigorous evaluation of these metrics allows for the development of reliable models that can be effectively applied to solve real-world problems and inform decision-making processes.

$$Sensitivity=\frac{TP}{TP+FN}*100$$

$$Specificity=\frac{TN}{TN+FP}*100$$

$$Accuracy\;(CCR\%)=\frac{TP+TN}{TP+TN+FP+FN}*100$$

SIMCA 14.1 software is commonly used to apply these techniques, offering robust tools for performing O2PLS-DA and HCA. SIMCA 14.1 facilitates the decomposition of data, visualization of class separation, and clustering of similar samples, thereby enhancing the analytical capabilities and interpretability of complex datasets in chemometric studies.

## 3. Results

From Table 1, there are significant differences in the percentage of volatile compounds found in each eucalyptus essential oil, which can be attributed to variations in soil, climate, and other environmental factors of the respective regions. Among the compounds analyzed, 1,8-Cineole (Eucalyptol) consistently displayed high mean values across all oils, with oil from Casablanca exhibiting the highest concentration (82.62 ± 0.87). This compound is known for its therapeutic properties and contributes to the characteristic aroma of eucalyptus essential oils. Significant variations were observed in the mean values of the compounds among the oils. For example, p-Cymene exhibited a significantly higher mean value in oils from Taounate (21.10 ± 6.16) and Tiflet (21.01 ± 3.42) compared to the other oils, indicating potential regional variations in the chemical composition of eucalyptus essential oils. Additionally, certain compounds showed consistent trends across multiple oils. Oil from Kenitra consistently displayed higher mean values for β-Pinene, α-Phellandrene, and Nerol compared to the other oils, suggesting a unique chemical profile for this particular oil. Interestingly, oil from Khemisset exhibited relatively low mean values for most compounds, particularly Sabinene, indicating a distinct chemical composition compared to the other oils. Overall, the comparison of volatile compounds among the six eucalyptus essential oils reveals variations in their chemical composition, indicating the presence of distinct chemotypes or regional variations. These findings have implications for the aroma, therapeutic properties, and potential applications of these oils in various industries, such as aromatherapy and pharmaceuticals.

### 3.1. O2PLS-DA Analysis of Volatile Compounds

**Figure 3:**
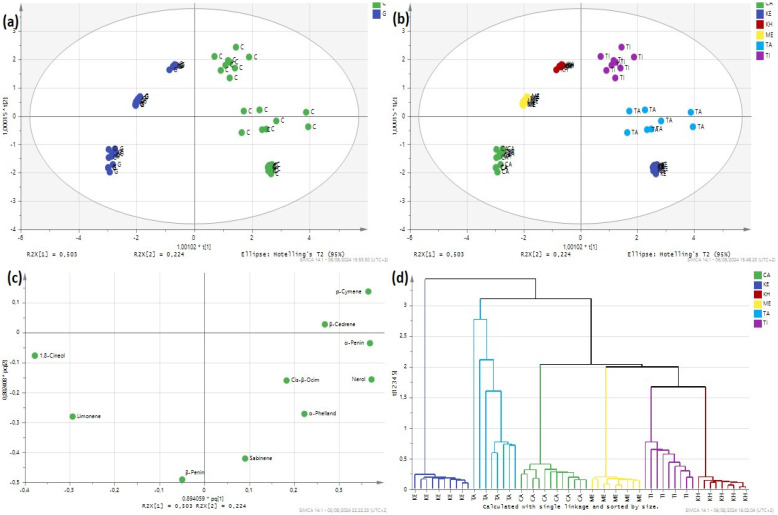
Comprehensive O2PLS-DA and HCA Analysis of Essential Oils: (**a**) Varietal Differentiation Score Plot, (**b**) Geographical Origin Score Plot, (**c**) Key Volatile Compound Loading Plot, and (**d**) HCA Dendrogram for Regional Classification (KE: Kenitra, TA: Taounate, TI: Tiflet, CA: Casablanca, KH: Khemisset, ME: Marrakech).

The application of Orthogonal Partial Least Squares Discriminant Analysis (O2PLS-DA) on the volatile composition of essential oils (EO) reveals profound insights into the classification and differentiation of these oils based on their varietal and geographical origins. O2PLS-DA, a powerful statistical technique, effectively categorizes EOs into two main groups: the globulus variety (Casablanca, Marrakech, and Khemisset) and the Camaldulensis variety (Kenitra, Bouzenika, Taounate, Tiflet). The score plot (Figure 3a) illustrates the geographical origin classification, showing a clear and well-defined separation of oils from different regions. The score plot (Figure 3a,b) indicates a clear and well-defined separation of oils within the globulus variety, suggesting that EOs from different geographical regions possess unique and identifiable characteristics. This geographic specificity is crucial for authenticating the origins of EOs, ensuring their quality and market value. The O2PLS-DA model exhibits remarkable accuracy, specificity, and sensitivity, all at 100%, demonstrating its robust predictive capability. The classification accuracy for each geographical region is detailed in Table 3, showcasing the model’s precision in distinguishing EOs based on their origin.

The high classification accuracy underscores the reliability of using volatile profiles as a fingerprinting approach for quality control. By analyzing the volatile composition, it becomes possible to accurately determine the origin and variety of an essential oil, ensuring its authenticity and adherence to specific quality standards. This is particularly important in the EO industry, where authenticity and quality are paramount for consumer trust and product efficacy.

The loading plot of the O2PLS-DA (Figure 3c) highlights the significant volatile compounds contributing to the differentiation between these groups. Notably, compounds such as 1,8 cineol and limonene are positively correlated with the oils from Casablanca, Marrakech, and Khemisset. These compounds are known for their distinctive aromatic properties and therapeutic benefits, making them key markers for identifying high-quality EOs from these regions. The high concentrations of 1,8 cineol and limonene in these samples suggest that these regions may have optimal growing conditions or specific cultivation practices that enhance the production of these compounds. Conversely, samples from Taounate, Kenitra, and Tiflet, which contribute negatively to the model, exhibit higher levels of nerol, penin alpha, and paracymene. The presence of these compounds in higher concentrations indicates a different volatile profile, which can be attributed to varying climatic conditions, soil types, or even genetic variations in the plants from these regions. The differentiation based on these volatile compounds is crucial for understanding the unique characteristics and potential applications of EOs from the Camaldulensis variety.

Hierarchical Cluster Analysis (HCA) in Figure 3d complements these findings by grouping the EOs into clusters based on their compositional similarities, further validating the distinctions observed in O2PLS-DA. HCA dendrograms reveal a clear separation between the different eucalyptus oils, with distinct sub-clusters corresponding to their specific regions. This clustering indicates that the compositional differences are consistent and robust, providing additional confidence in the classification model. The clear separation in HCA also suggests the potential for developing targeted marketing strategies for EOs based on their regional characteristics, thereby enhancing their commercial value.

Moreover, the findings highlight the potential for using O2PLS-DA and HCA in other applications within the EO industry. For instance, these techniques could be employed to detect adulteration, monitor changes in EO composition due to environmental factors, or even guide breeding programs aimed at enhancing specific volatile compounds. The ability to differentiate EOs based on their volatile profiles also opens up possibilities for creating customized blends tailored to specific therapeutic or aromatic preferences, further expanding the market potential of these natural products.

The validation results obtained using volatile profile data from GC–FID (Table 2) underscore the robustness and precision of the O2PLS-DA model in classifying essential oils (EOs) based on both geographical origin and varietal characteristics. The model demonstrated a perfect classification accuracy of 100% across all samples, effectively distinguishing EOs from Kenitra, Taounate, Tiflet, Casablanca, Khemisset, and Marrakech. Each sample was correctly assigned to its respective geographical origin and varietal group, highlighting the distinct volatile profiles unique to each region. For instance, samples from Kenitra, Taounate, and Tiflet (Camaldulensis variety) were accurately classified due to their higher concentrations of nerol, penin alpha, and paracymene. Conversely, samples from Casablanca, Marrakech, and Khemisset (globulus variety) were distinguished by higher levels of 1,8 cineol and limonene. The Fisher’s probability value of 1.5 × 10^−11^ indicates a highly significant difference between the groups, reinforcing the model’s validity. The specificity and sensitivity of the O2PLS-DA model were both 100%, underscoring its exceptional capability in correctly identifying and distinguishing the essential oils without any false positives or negatives. These results affirm the utility of volatile profiles as a reliable fingerprinting analytical method for EO authentication and quality control, ensuring product integrity and enhancing market value.

### 3.2. O2PLS-DA Analysis of Eucalyptus Essential Oil Spectroscopic Data

**Figure 4:**
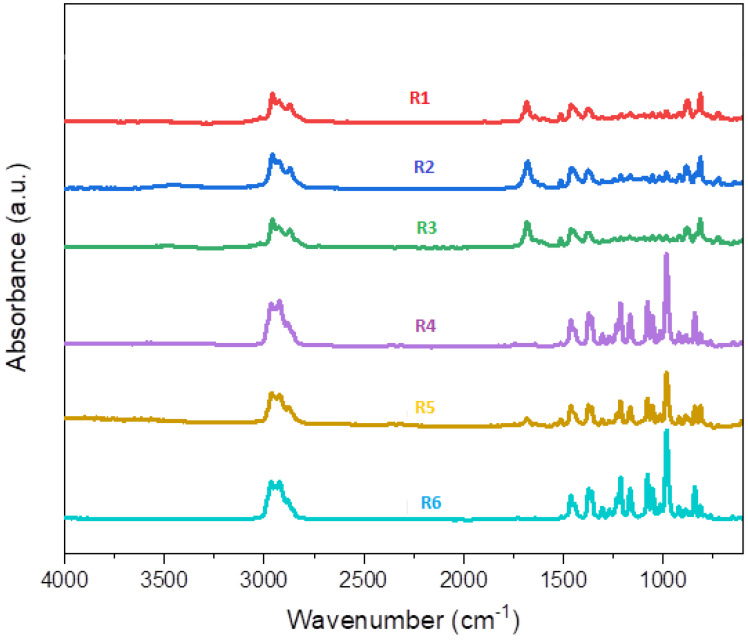
FTIR Spectral Analysis of Essential Oils: Comparison of Spectral Profiles for Camaldulensis and Globulus Varieties (R1: Kenitra, R2: Taounate, R3: Tiflet for Camaldulensis; and R4: Casablanca, R5: Khemisset, R6: Marrakech for Globulus).

The FTIR spectrum analysis of the essential oil (EO) in Figure 4 revealed distinctive absorption bands corresponding to its major components. Among the identified compounds is 1,8-cineol, a key active ingredient responsible for the insecticidal properties of Eucalyptus oil. Specific absorption bands associated with this bicyclic compound include a prominent peak at 985 cm^−1^, indicating symmetrical bending out of the CH₂ plane. Additionally, bands at 1079 and 1214 cm^−1^ are attributed to the symmetric and asymmetric stretches of the C–O–C group, respectively. Another significant absorption band at 1374 cm^−1^ corresponds to CH₃ deformation, while a characteristic absorption at 1377 cm^−1^ is linked to the deformation of the C–O–H group.

The FTIR spectrum (Figure 4) also displayed common absorption bands indicative of various functional groups present in the EO, including double bonds, methyl, and methylene groups. Bands at 2957 and 2870 cm^−1^ correspond to the symmetric and asymmetric stretches of CH_3_, while the band at 2923 cm^−1^ is associated with the asymmetric stretch of CH₂. An absorption at 1642 cm^−1^ signifies the stretching vibrations in C=C bonds of olefinic compounds. Furthermore, a strong absorption at 1456 cm^−1^ is linked to the symmetrical bending of C–H bonds within the molecular plane. The absence of bands in the range of 3200–3600 cm^−1^, typical for OH groups, suggests low concentrations of compounds such as citronellol, isopulegol, and neo-isopulegol in the isolated essential oil.

The peak intensities and patterns of the R4, R5, and R6 spectra closely align with the infrared spectrum of 1,8-cineol, as depicted in the Spectral Database for Organic Compounds. These three groups of essential oils are characterized by a high content of cineol. By carefully comparing the six FTIR mean spectra, focusing on peak positions, intensities, and spectral patterns, it becomes evident that the spectra are divided into two distinct groups: the first group, consisting of R1, R2, and R3, corresponds to Camaldulensis variety, while the second group, consisting of R4, R5, and R6, corresponds to globulus variety. The main differences between these species are observed in the region between 1000 and 1500 cm^−1^, highlighting significant variations in their spectral profiles.

This detailed FTIR analysis provides valuable insights into the chemical composition, functional groups, and structural differences between the two species under investigation. Such information is crucial for selecting the appropriate oil for specific therapeutic applications, such as aromatherapy, skincare, and medicinal purposes. However, visual observation of the spectra for samples belonging to the same species does not allow for their classification based on geographical origin. Therefore, multivariate analysis tools have been applied to study the similarities between samples and evaluate the capability of FTIR to discriminate between them based on their geographical origin.

**Figure 5:**
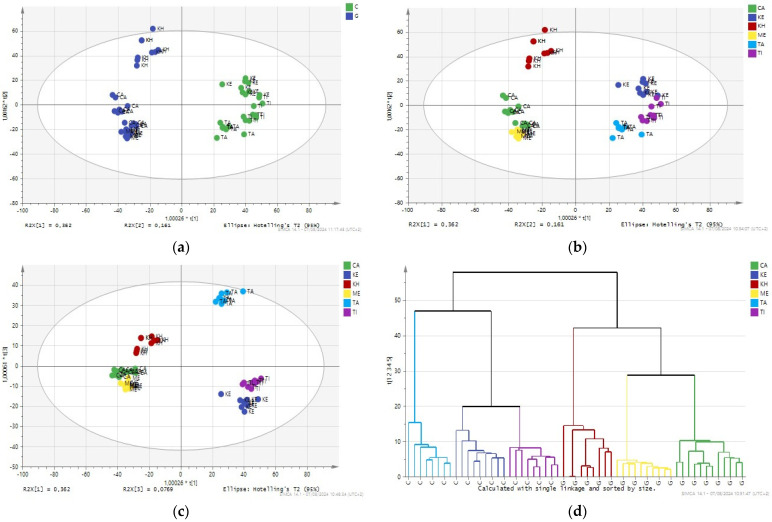
Classification and Clustering of Eucalyptus Essential Oils: O2PLS-DA and HCA Analysis. (**a**): Classification of Eucalyptus Essential Oils by Variety: LV1 vs. LV2 Plot, (**b**): Geographical Origin Classification of Eucalyptus Essential Oils: LV1 vs. LV2 Plot, (**c**): Geographical Origin Classification of Eucalyptus Essential Oils: LV1 vs. LV3 Plot, and (**d**): Hierarchical Cluster Analysis (HCA) of Eucalyptus Essential Oils (KE: Kenitra, TA: Taounate, TI: Tiflet, CA: Casablanca, KH: Khemisset, ME: Marrakech).

The O2PLS-DA (Orthogonal Projections to Latent Structures Discriminant Analysis) model was applied directly to raw FTIR (Fourier Transform Infrared) spectroscopy data, without additional spectral preprocessing, to provide a comprehensive and detailed classification of Eucalyptus essential oils from different varieties and geographical origins. The analysis includes two primary plots, LV1 vs. LV2 and LV1 vs. LV3, which collectively offer profound insights into the chemical diversity and origin-based classification of the essential oils, as shown in Figure 5b,c. The LV1 vs. LV2 plot demonstrates a remarkable ability to distinguish between the two Eucalyptus varieties, Camaldulensis and Globulus, with perfect separation of samples from various origins (Figure 5a). This indicates that the primary and secondary principal components capture the essential variations in chemical composition attributable to both variety and geographical location. The LV1 vs. LV3 plot (Figure 5c) further substantiates these findings, showing consistent separation and reinforcing the reliability of the O2PLS-DA model. The consistency across different principal component pairings underscores the robustness of the classification and the inherent chemical differences between the samples.

The performance metrics in Table 3 showcase the exceptional accuracy of the O2PLS-DA model, with each sample correctly classified according to its geographical origin with a 100% success rate. Specifically, all 9 samples from Kenitra (R1) are correctly assigned to class KE, all 8 samples from Taounate (R2) to class TA, all 9 samples from Tiflet (R3) to class TI, all 12 samples from Casablanca (R4) to class CA, all 9 samples from Khemisset (R5) to class KH, and all 10 samples from Marrakech (R6) to class MA. These results indicate that the model achieves flawless classification, with no misclassified samples or unassigned predictions. The comprehensive classification underscores the distinctiveness of the chemical profiles associated with each geographical origin. The Fisher’s probability value of 5 × 10^−40^ highlights the extreme statistical significance of the classification results. This value indicates that the probability of achieving such a perfect classification by chance is astronomically low, thereby affirming the reliability and robustness of the O2PLS-DA model. The highly significant Fisher’s probability demonstrates the effectiveness of the model in capturing the underlying chemical distinctions between the samples. The O2PLS-DA-HCA (Hierarchical Cluster Analysis) in Figure 5d further validates the classification results by grouping samples with similar chemical profiles together. HCA provides an additional layer of verification, demonstrating consistent clustering of samples according to their origins. This complementary analysis supports the findings from the O2PLS-DA plots and performance metrics, providing a holistic view of the classification.

### 3.3. Validation Set Results

The performance metrics in Table 4 for the validation set further support the high accuracy of the O2PLS-DA model, with a 95.83% overall success rate in classifying samples according to their geographical origin. Specifically, all samples from Kenitra, Taounate, Casablanca, Khemisset, and Marrakech are correctly classified with 100% accuracy. For Tiflet (TI), the model correctly classifies two out of three samples, resulting in a 66.67% accuracy rate. This slight deviation indicates that while the model performs exceptionally well, there is less room for improvement in handling some specific samples. The comprehensive classification underscores the distinctiveness of the chemical profiles associated with each geographical origin.

In terms of variety classification, the model shows robust performance. For the varieties Camaldulensis and Globulus, the O2PLS-DA model achieves 100% CCR, 100% specificity, and 100% sensitivity. These metrics highlight the model’s ability to accurately discriminate between the two varieties based on their chemical composition.

The Fisher’s probability value of 1.8 × 10^−10^ highlights the extreme statistical significance of the validation results. This value indicates that the probability of achieving such a high classification accuracy by chance is astronomically low, thereby affirming the reliability and robustness of the O2PLS-DA model. The highly significant Fisher’s probability demonstrates the effectiveness of the model in capturing the underlying chemical distinctions between the samples.

## 4. Discussion

This study’s comprehensive analysis of Eucalyptus essential oils (EOs) using FTIR spectroscopy, volatile compound profiling, and chemometric methods has yielded significant insights into the geographical and varietal differentiation of EOs. Our findings align with and expand upon the existing body of research in this domain, especially as the first study of its kind conducted in Morocco, adding a novel contribution to the global understanding of Eucalyptus EOs.

Previous studies have documented the impact of geographical origin and species variety on the chemical composition of Eucalyptus EOs. For instance, refs. [4,22] reported that the concentration of major components like 1,8-cineole, alpha-pinene, and limonene varies significantly across different Eucalyptus species and geographical locations. Our results corroborate these findings, showing that oils from Casablanca, Marrakech, and Khemisset (E. globulus) have higher concentrations of 1,8-cineole and limonene, while oils from Kenitra, Taounate, and Tiflet (E. camaldulensis) exhibit higher levels of nerol, alpha-pinene, and p-cymene.

Additionally, findings from a recent study align with our observations regarding the effect of geographical origin and botanical variety on the chemical composition and extraction yield of Eucalyptus Eos [16]. This study used chemometric techniques, specifically Principal Component Analysis (PCA), to show that EO composition depends predominantly on these two factors, underscoring the significant role of geographical and varietal differences in EO profiling. Importantly, this study highlighted that FT-MIR spectroscopy, in combination with partial least squares regression (PLS-R), could accurately predict the chemical composition of Eucalyptus EOs, offering a potential alternative to the GC–FID method. This is consistent with our findings, as FTIR spectroscopy effectively distinguished between the spectral profiles of E. globulus and E. camaldulensis, indicating the promising role of spectroscopic techniques in rapid EO classification and chemical prediction.

The use of FTIR spectroscopy to differentiate EOs based on their chemical composition has been well-documented in literature. Studies have demonstrated the efficacy of FTIR in identifying and quantifying key functional groups and compounds in essential oils [23,24]. Our FTIR analysis aligns with these studies, identifying distinct absorption bands corresponding to major components like 1,8-cineole, and highlighting significant spectral differences between E. globulus and E. camaldulensis [25].

Chemometric methods, particularly O2PLS-DA and HCA, have been increasingly employed to classify EOs based on their chemical profiles [15]. Research has shown that these techniques can effectively differentiate EOs from various geographical regions and species [25,26,27]. Our study achieved 100% classification accuracy using O2PLS-DA, demonstrating the robust predictive capability of this method. The clear separation observed in HCA further validates the distinct chemical profiles of EOs from different origins.

The geographical differentiation observed in our study is consistent with findings from other studies, which reported significant regional variations in the chemical composition of Eucalyptus EOs [4,28]. The high concentrations of 1,8-cineole in oils from Casablanca and Marrakech suggest optimal growing conditions for this compound in these regions, similar to observations in Australian Eucalyptus species [29]. Our results underscore the importance of volatile profiling and FTIR spectroscopy in the quality control and authentication of Eucalyptus EOs. The ability to accurately classify EOs based on their geographical origin and variety has significant implications for the industry, ensuring product consistency and protecting against adulteration. These findings are in line with conclusions from studies [8,30,31], which emphasize the need for robust analytical methods to maintain EO quality and authenticity.

This study is the first to investigate the volatile composition and FTIR spectral characteristics of Eucalyptus EOs specifically from Morocco. By providing a detailed chemical and spectral profile of EOs from different Moroccan regions and varieties, this research adds a novel contribution to the existing body of knowledge. The findings highlight the unique characteristics of Moroccan Eucalyptus EOs, setting a foundation for future research and applications in the EO industry. Furthermore, the integration of spectroscopic techniques like FTIR with chemometric models, as demonstrated in our study and corroborated by recent findings, presents a promising path for rapid, reliable EO characterization based on geographical origin and botanical variety.

One limitation of our study lies in the timing and scope of sampling. All samples analyzed in this study were collected within the same year, specifically between September and October 2022. This timeframe may not capture potential seasonal variations in the chemical composition of Eucalyptus essential oils, which could influence the generalizability of our findings across different harvest periods. Future studies that expand the sampling timeframe across multiple seasons and years could provide additional insights into the temporal stability of the observed chemical and spectral profiles. Despite this limitation, the current study establishes a foundational understanding of Moroccan Eucalyptus EOs and demonstrates the utility of FTIR spectroscopy combined with chemometric methods for effective classification by geographical origin and variety.

## 5. Conclusions

This pioneering study represents the first comprehensive analysis of Eucalyptus essential oils (EOs) in Morocco, utilizing FTIR spectroscopy, volatile compound profiling, and advanced chemometric techniques. Significant differences were identified in the volatile profiles of EOs from Eucalyptus globulus and Eucalyptus camaldulensis across six Moroccan regions. FTIR spectroscopy effectively distinguished EOs based on their chemical composition, while O2PLS-DA and HCA methods provided accurate classification by geographical origin and species variety, achieving 100% classification accuracy. These findings highlight the importance of combining analytical and chemometric methods for quality control and authentication in the EO industry. The study not only expands the global understanding of EO composition and variability but also emphasizes the unique characteristics of Moroccan Eucalyptus EOs, providing a foundation for future research and applications.

## Figures and Tables

**Table 1:** Comparison of the main total volatile compounds in extracts of the seven eucalyptus essential oils (results given as mean ± SD; for each region). Classes with the same superscript letter are not significantly different from each other according to multiple pairwise comparisons at α = 0.05.

Region	*1,8-Cineole*	*β-Pinene*	*Limonene*	*p-Cymene*	*Cis-β-Ocimene*	*Sabinene*	*α-Phellandrene*	*Nerol*	*α-Pinene*	*β-Cedrene*
**Casablanca**	82.62 ± 0.87 ^a^	2.23 ± 0.29 ^a^	6.49 ± 0.36 ^a^	4.47 ± 0.34 ^c^	2.58 ± 0.22 ^d^	0.42 ± 0.04 ^b^	0.81 ± 0.06 ^d,e^	0.11 ± 0.04 ^e^	0.00 ± 0.00 ^d^	0.00 ± 0.00 ^d^
**Taounate**	6.07 ± 0.69 ^f^	1.16 ± 0.39 ^b,c^	1.85 ± 0.50 ^c^	21.10 ± 6.16 ^a^	6.99 ± 0.75 ^b^	0.60 ± 0.31 ^a^	0.50 ± 0.15 ^e,f^	1.56 ± 0.20 ^b^	1.79 ± 0.43 ^b^	1.35 ± 1.36 ^a^
**Kenitra**	20.10 ± 0.21 ^c^	2.11 ± 0.11 ^a^	1.62 ± 0.07 ^c,d^	18.64 ± 0.27 ^a^	3.10 ± 0.11 ^c^	0.62 ± 0.05 ^a^	8.53 ± 0.09 ^a^	1.90 ± 0.07 ^a^	2.21 ± 0.02 ^a^	0.56 ± 0.02 ^b,c^
**Khemisset**	56.41 ± 2.62 ^b^	0.72 ± 0.02 ^c^	1.95 ± 0.21 ^c^	13.32 ± 1.12 ^b^	2.11 ± 0.05 ^e^	0.00 ± 0.00 ^d^	1.48 ± 0.12 ^c^	0.49 ± 0.01 ^d^	0.59 ± 0.02 ^c^	0.16 ± 0.02 ^c,d^
**Marrakech**	81.87 ± 0.61 ^a^	1.52 ± 1.10 ^b^	2.78 ± 1.20 ^b^	7.22 ± 1.04 ^c^	2.34 ± 0.60 ^d,e^	0.42 ± 0.05 ^b^	0.01 ± 0.03 ^f^	0.13 ± 0.02 ^e^	0.00 ± 0.00 ^d^	0.00 ± 0.00 ^d^
**Tiflet**	10.29 ± 0.76 ^e^	0.96 ± 0.19 ^b,c^	1.04 ± 0.09 ^d^	21.01 ± 3.42 ^a^	1.42 ± 0.05 ^f^	0.09 ± 0.03 ^c,d^	1.30 ± 0.64 ^c,d^	0.77 ± 0.13 ^c^	1.77 ± 0.50 ^b^	0.77 ± 0.07 ^a,b^

**Table 2:** Confusion Matrix Analysis of volatile compounds in Essential Oil Validation Sample Classification by Geographic Origin and Botanical Variety with Sensitivity and Specificity Metrics.

Origin	Variety	KE	TA	TI	CA	KH	ME	Sensitivity%	Specificity%	CCR%
KE	Camaldulensis	3	0	0	0	0	0	100	100	100
TA		0	3	0	0	0	0	100	100	100
TI		0	0	3	0	0	0	100	100	100
CA	Globulus	0	0	0	4	0	0	100	100	100
KH		0	0	0	0	3	0	100	100	100
ME		0	0	0	0	0	4	100	100	100
Total		3	3	3	4	3	4	100	100	100
Fisher’s prob.		1.5 × 10^−11^								

**Table 3:** Performance Metrics and Classification Accuracy of O2PLS-DA Model for Eucalyptus Essential Oils.

Origin	Variety	KE	TA	TI	CA	KH	ME	Sensitivity%	Specificity%	CCR%
KE	Camaldulensis	9	0	0	0	0	0	100	100	100
TA		0	8	0	0	0	0	100	100	100
TI		0	0	9	0	0	0	100	100	100
CA	Globulus	0	0	0	12	0	0	100	100	100
KH		0	0	0	0	9	0	100	100	100
ME		0	0	0	0	0	10	100	100	100
Total		9	8	9	12	9	10	100	100	100
Fisher’s prob.		5 × 10^−40^								

**Table 4:** Performance Metrics for O2PLS-DA Model Validation.

Origin	Variety	KE	TA	TI	CA	KH	ME	Sensitivity%	Specificity%	CCR%
KE	Camaldulensis	3	0	0	0	0	0	100	100	100
TA		0	3	0	0	0	0	100	94.12	75.00
TI		0	1	2	0	0	0	66.67	100	66.67
CA	Globulus	0	0	0	4	0	0	100	100	100
KH		0	0	0	0	3	0	100	100	100
ME		0	0	0	0	0	4	100	100	100
Total		3	3	3	4	3	4	94.44	99.02	95.83
Fisher’s prob.		1.8 × 10^−10^								

## Data Availability

The data that support the findings of this study are available on request from the corresponding author.
